# Dexamethasone enhances programmed cell death 1 (PD-1) expression during T cell activation: an insight into the optimum application of glucocorticoids in anti-cancer therapy

**DOI:** 10.1186/s12865-015-0103-2

**Published:** 2015-06-26

**Authors:** Kailin Xing, Bingxin Gu, Ping Zhang, Xianghua Wu

**Affiliations:** Department of Medical Oncology, Fudan University Shanghai Cancer Center, 270 Dong-An Road, Shanghai, 200032 China; Department of Oncology, Shanghai Medical College, Fudan University, 130 Dong-An Road, Shanghai, 200032 China; Department of Nuclear Medicine, Fudan University Shanghai Cancer Center, 270 Dong-An Road, Shanghai, 200032 China; Cancer Research Institute of Fudan University Shanghai Cancer Center, 270 Dong-An Road, Shanghai, 200032 China

**Keywords:** PD-1, Glucocorticoids, Naïve T cells, Memory T cells

## Abstract

**Background:**

Programmed cell death 1 (PD-1) is a key cell-surface receptor of CD28 superfamily that triggers inhibitory pathways to attenuate T-cell responses and promote T-cell tolerance. As a crucial role in tumor immunity, PD-1 has been a focus of studies in anti-cancer therapy. It has been approved that tumors could exploit PD-1-dependent immune suppression for immune evasion. Considering the wide use of glucocorticoids (GCs) in anti-cancer therapy and their immunosuppressive effects, we explored whether GCs could influence the expression of PD-1.

**Results:**

In our study, we used dexamethasone (DEX) as a model glucocorticoid and demonstrated that DEX could enhance PD-1 expression in a dose-dependent manner. The effects were completely inhibited by the glucocorticoid receptor (GR) antagonist mifepristone (RU486), indicating that the effect of DEX on PD-1 is mediated through GR. We further found the sensitivity to DEX-induced upregulation of PD-1 expression had a significant difference between different T cell subsets, with memory T cells more susceptible to this effect. We also showed that DEX could suppress T cell functions via inhibition of cytokines production such as IL-2, IFN-γ, TNF-α and induction of apoptosis of T cells.

**Conclusion:**

Our findings suggest a novel way by which DEX suppress the function of activated T lymphocytes by enhancing expression of PD-1 and provide an insight into the optimum clinical application of GCs.

## Background

Glucocorticoids (GCs) have been widely used as immunosuppressive and anti-inflammatory agents in the treatment of numerous autoimmune and inflammatory diseases. They also play an important role in anti-cancer therapy. The potent capacity to kill lymphoid cells has led to their inclusion in all chemotherapy protocols for lymphoid malignancies [1], e.g., prednisone combined with rituximab, cyclophosphamide, doxorubicin and vincristine (R-CHOP) acts as the standard first-line therapy for diffuse large B-cell lymphoma (DLBCL). Besides, GCs are widely used as co-medication in cancer therapy of solid malignant tumors to prevent chemotherapy-related hypersensitivity reactions and other adverse effects such as nausea, emesis and toxic reactions [2, 3]. Before, during and after chemotherapy of solid malignant tumors, GCs are given at varying doses to reduce acute toxicity or to protect normal tissue [4].

However, accumulating evidences show that the application of GCs may interfere with the therapeutic efficacy in anti-cancer therapy. It renders the majority of malignant tumor cells resistant to apoptosis and promotes proliferation in several established and primary carcinoma cancer cells [5, 6]. Dexamethasone (DEX), an important member of glucocorticoid family, has been approved to antagonize the efficacy of some anti-cancer drugs such as cisplatin, 5-fluorouracil, actinomycin D, doxorubicin and gemcitabine [7, 8]. In addition, M. Xia found that DEX could enhance the expression of cytotoxic T-lymphocyte-associated antigen (CTLA)-4 during T cell activation [9], indicating that DEX might suppress antitumor immune responses and facilitate tumor progression.

Similar to CTLA-4, programmed cell death 1 (PD-1) is a key cell-surface receptor of CD28 superfamily that triggers inhibitory pathways and dampens T-cell activity when bound by its ligands, PD-L1 or PD-L2 [10]. Studies have shown that PD-1 is highly expressed on tumor infiltrating lymphocytes and PD-L1 is commonly up-regulated on many different tumor types, resulting in the inhibition of local anti-tumor T cell responses [11]. Cancers can elude immune surveillance through PD-1/PD-L1 pathway and pre-clinical studies demonstrated that high levels of expression of PD-L1 correlates with poor prognosis of cancer [12–14]. However, it still remains unclear how the expression of PD-1 and its ligands are spatially and temporally regulated and what are the molecular mechanisms of signaling through PD-1 and its ligands. Based on the evidence that DEX could enhance CTLA-4 expression [9], we explored whether DEX could enhance PD-1 expression as well. Our results demonstrated that DEX could also enhance the expression of PD-1 both in mouse and human activated T cells dose-dependently, which was mediated by glucocorticoid receptor (GR).

## Methods

### Animals

Female Balb/c mice of 6–8 weeks of age were purchased from Ling Chang Corporation (Shanghai, China). All mice were kept under specific pathogen-free conditions in the Experimental Animal Center of Fudan University. All of the experiments were approved by the Institutional Animal Care and Use Committee of Fudan University.

### Mouse T-cell isolation and sorting

Mouse lymphocytes were harvested from spleens of Balb/c mice by grinding tissue through sterile wire mesh. T cells were purified by the Pan T Cell Isolation Kit by using EasySep beads (STEMCELL Technologies Inc., Canada) following manufacturer's instructions. Postsorting analysis of purified subsets revealed greater than 95 % purity.

### Human T-cell isolation and sorting

Peripheral blood mononuclear cells (PBMCs) were harvested from healthy donors after informed consent. This study has been approved by the Ethics Committee of Fudan University Shanghai Cancer Center. PBMCs were isolated by Ficoll-Paque PLUS (GE Healthcare), and enriched for CD3+ cells with the Pan T Cell Isolation Kit by using EasySep beads (STEMCELL Technologies Inc., Canada) following the manufacturer’s instructions. Postsorting analysis of purified subsets revealed greater than 95 % purity.

### Cell culture and treatment

Sorted T cells were resuspended in RPMI1640 medium containing 10 % heat inactivated fetal bovine serum and 2 mM L-glutamine (Gibco). Cells were cultured in 24-well plates and stimulated with anti-CD3/CD28 coated beads following manufacturer’s instructions. Beads were removed before flow cytometry. DEX (10^−9^–10^−5^ M), RU486 (10^−9^–10^−5^ M) were added as treatment groups. The media (with fresh DEX or RU486 as appropriate) were changed every 3 days. Cells were incubated at 37 °C in 5 % CO_2_ before being collected and analyzed by flow cytometry.

### Reagents

DEX was purchased from Sigma. Hydrocortisone was from Tianjin Biochem Pharmaceutical Cooperation (China), Mifepristone (RU486) was from Tokoyo Chemical Industry (TCI, Japan). Antibodies for flow cytometry, including Percp-cy5.5 labeled anti-mouse or human CD3, Alexa Fluor 700 labeled anti-mouse or human CD4, APC labeled anti-mouse or human CD8, FITC labeled anti-mouse CD44, PE labeled anti-mouse CD62L, APC or PE labeled anti-mouse or human PD-1 and PD-1 isotype were all purchased from Biolegend. Mouse or human T-activator CD3/CD28 beads were obtained from Gibco (Invitrogen Life Technologies). Mouse or human Pan T Cell Isolation Kit were from STEMCELL Technologies Inc.

### Flow cytometry analysis

Cultured T cells were collected at indicated time after stimulated by anti-CD3/CD28 beads at 1:1 bead-to-cell ratio and analyzed by flow cytometry. Cells were washed with PBS twice and incubated for 20 min at room temperature in cell staining buffer (Biolegend) with different combinations of the surface staining antibodies. After washing with PBS twice, the stained cells were analyzed on Beckman Counter FC500. All data were analyzed with FlowJo software (Tree Star, Inc.).

### Quantitative Real-time PCR

Sorted mouse T cells were activated and treated with or without DEX (1 × 10^−7^ M). RNA was extracted after 48 h of the treatment using Trizol Reagent (Invitrogen) and subjected to reverse transcription using PrimeScript RT reagent Kit (Takara Bio Inc., Japan). After quantified by spectrophotometry, Real-time (RT)-PCR amplification was performed with a Mastercycler® pro (Eppendorf, Hamburg, Germany) using SYBR Green (Takara Bio Inc., Japan) under the following conditions: 95 °C for 30 s followed by 40 cycles of 95 °C for 5 s, 62 °C for 34 s and a melting curve collection. The primer sets used for amplification were: PD-1-F: 5'-TGCTCAACAAGTATGTCAGAGG-3'; PD-1-R: 5'-ACACTAGGGACAGGTGCTGC-3'; β-actin-F: 5'-AGAGGGAAATCGTGCGTGAC-3'; β-actin-R: 5'-CAATAGTGATGACCTGGCCGT-3'. β-actin was used in parallel reactions as internal control. Statistical analyses were performed using the 2^-ΔCT^ relative quantification method.

### Western blot analysis

Sorted mouse T cells were activated and treated with or without DEX (1 × 10^−7^ M). After 48 h, cells were collected and lysed in RIPA buffer supplemented with complete protease inhibitor cocktail (Roche, Basel, Switzerland). Protein concentrations were determined using the BCA protein assay kit (Biyotime, Shanghai, China). Cell lysates (20 μg) were separated by SDS-PAGE and transferred into polyvinylidene fluoride (PVDF). The membrane was then blocked with 5 % skim milk in PBST (0.29 % Na_2_HPO_4_, 0.8 % NaCl, 0.02 % KCl, and 0.05 % Tween-20, pH7.4) for 2 h at room temperature and then incubated with anti-PD-1 mAb (Abcam) at 4°Covernight. After washing three times with PBST, the membrane was incubated with HRP-conjugated secondary antibody (Immunology Consultants Laboratory, Inc.) and detected by enhanced chemiluminescence reagent (Pierce, Rockford, IL, USA). Anti-β-actin mAb (Cell Signaling Technology, USA) was used as internal standard.

### ELISAs for cytokine production

The culture supernatants of mouse activated T cells were removed 48 h after different treatments and kept at −80 °C until cytokine measurement. IL-2, TNF-α and IFR-γ productions were measured using quantitive immunoassay ELISA kits (R&D Systems, Minneapolis, MN, USA) in 96-well microtiter plates according to the manufacturer’s instructions. The concentration of the cytokines in each sample was obtained by extrapolation from a standard calibration curve generated simultaneously. Results were expressed as pg/mL.

### Analysis of apoptosis

Mouse activated T cells were cultured in the absence or presence of DEX (1 × 10^−7^ M). at different time points (6, 12, 24, and 48 h), percentage of apoptosis was determined by using FITC-labelled Annexin V and propidium iodide (PI) (Biolegend). Briefly, 1 × 10^6^ cells were resuspended in 100 μL of binding buffer (Biolegend) containing annexin V and PI according to manufacturer’s instructions. After incubation for 15 min in the dark at room temperature, cells were analyzed with a Beckman Counter FC500 using FlowJo software.

### Statistical analysis

Statistical analysis was performed using GraphPad Prism 5.0 software. Data are expressed as the mean ± SEM. The statistical significance of differences was evaluated by Student’s *t*-test. Values of P < 0.05 were considered to be significant.

## Results

### Kinetics of cell surface PD-1 expression during T cell activation

**Fig. 1:**
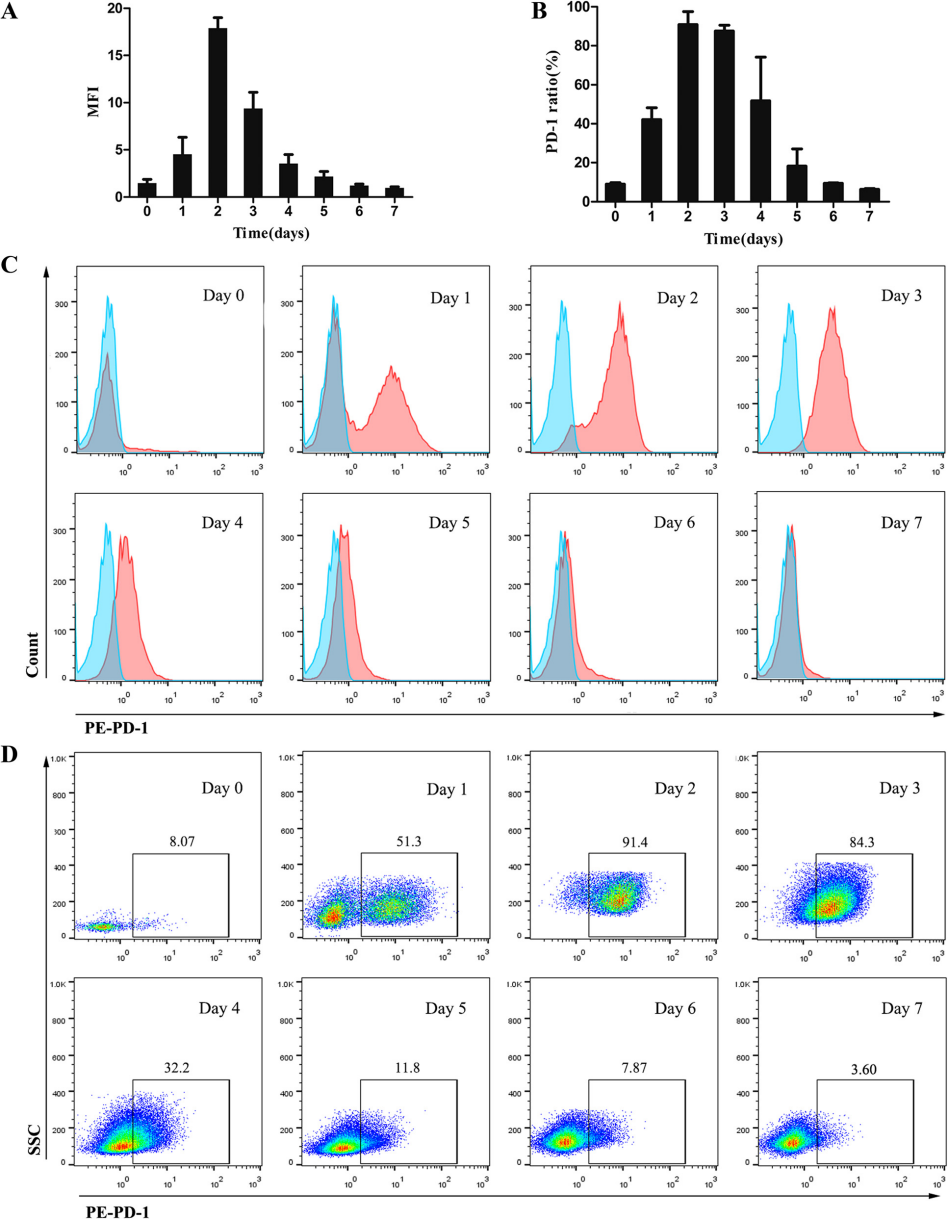
Kinetics of surface PD-1 expression of T lymphocytes during activation. Cells were double-stained with Percp-cy5.5 labeled anti-CD3 antibody and either PE-labeled isotype control or anti-PD-1 antibody. **a** Dynamic variation of MFI of PD-1 expression on mouse CD3^+^ T cells after activation. **b** Dynamic variation of ratio of PD-1 expression on mouse CD3^+^ T cells after activation. **c** Representative flow cytometry histograms of PD-1 expression (red) and isotype control (cyan) gated on CD3^+^ T cells from day 1 to day 7 after activated (day 0 represent the baseline). **d** Representative dot plots showing the percentage of T cells expressing PD-1 from day 1 to day 7 after activated (day 0 represent the baseline). Data shown are representative of three independent experiments

PD-1 is an inducible costimulatory molecule expressed on T cells, B cells, natural killer (NK) cells, dendritic cells (DCs) and monocytes upon activation [15–17]. Previous study has examined that PD-1 is not expressed on resting T cells but is inducibly expressed within 24 h after stimulation [18]. As an initial step to understand the basis for the effect of DEX on PD-1, we first analyzed the dynamic changes of PD-1 expression on activated T cells. Freshly isolated mouse splenocytes without stimulation were analyzed by flow cytometry. Data showed there is almost no expression on resting CD3-positive T cells (Fig. 1 Day 0). Then cells were stimulated with anti-CD3/CD28 beads at 1:1 bead-to-cell ratio for 1-7d. As shown in Fig. 1, both the mean fluorescence intensity (MFI) and proportion of PD-1^+^ T cells were up-regulated within 24 h and reached a peak at 48 h, followed by reduction in the next days. It returned to background levels by day 7. On the basis of these findings, we chose 48 h as our next observation time point.

### DEX enhances PD-1 expression both in mouse and human activated T cells

**Fig. 2:**
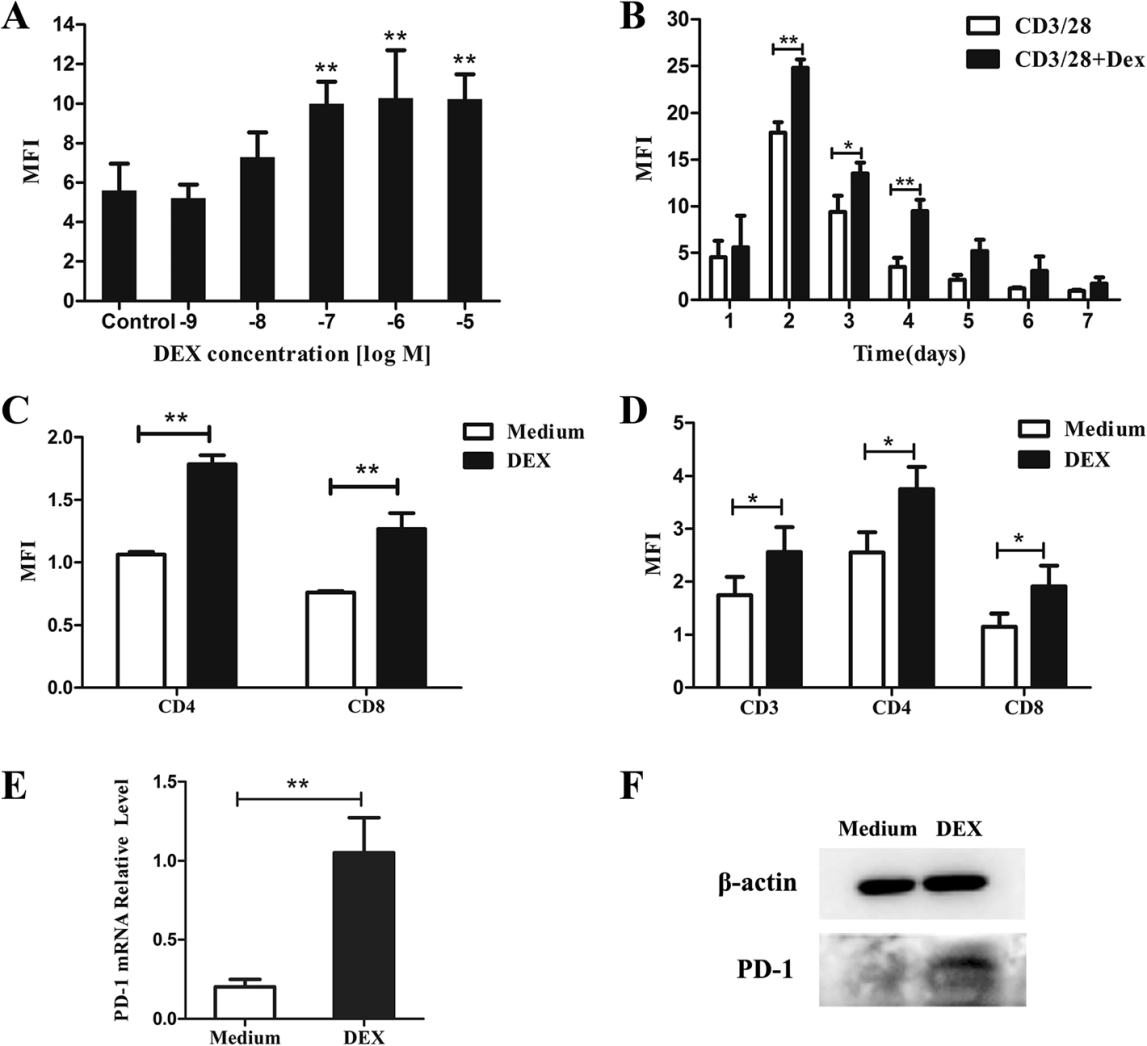
Effects of DEX on PD-1 expression on activated T cells. **a** Mouse T cells were incubated with anti-CD3/CD28 beads for 48 h in the absence or presence of various doses of DEX (10^−9^-10^−5^ M). **b** Mouse T cells were incubated with anti-CD3/CD28 beads in the absence or presence of 1 × 10^−7^ M DEX for 0 to 7 days. DEX significantly increased PD-1 expression. **c** DEX increased PD-1 expression in both CD4^+^ and CD8^+^ subsets of mouse T cells. **d** DEX increased PD-1 expression in both CD4^+^ and CD8^+^ subsets of human T cells. **e** Analysis of PD-1 mRNA level in DEX-treated and untreated T cells by real-time PCR analysis. Data are presented as the mean fold change in PD-1 mRNA level normalized to the β-actin mRNA. **f** Western blot analyses confirming higher expression of PD-1 protein in DEX-treated T cells. Data represents mean ± SEM of three experiments. *p < 0.05. **p < 0.01

To investigate the effects of DEX on PD-1 expression in mouse T cells after activation, DEX at different doses (10^−9^ to 10^−5^ M) were added to culture medium at the same time with activation. Forty-eight hours later, cells were collected for flow cytometry. Results suggest that DEX dose-dependently enhanced the expression of PD-1 at 48 h with EC_50_ values of 10^−7^ M (Fig. 2a). Though data showed DEX at 10^−9^ M slightly reduced the expression than control, this difference has no significance (P > 0.05) (Fig. 2a). To further explore whether this effect exists during whole activation, mouse T cells were incubated with anti-CD3/CD28 beads in the absence or presence of 1 × 10^−7^ M DEX from day 1 to day 7. We found DEX enhanced PD-1 expression at each time point, with difference more significantly from day 2 to day 4 (p < 0.05) (Fig. 2b). These results confirmed our hypothesis that DEX could enhance PD-1 expression during T cell activation. We also observed that surface expression of PD-1 was upregulated in both CD4+ and CD8+ T cells (Fig. 2c). Besides, our results revealed that DEX did not induce PD-1 expression in unstimulated T cells (data not shown).

To ascertain whether DEX exerts similar effect on human T cells, we treated activated human T cells with DEX (10^−7^ M). The flow cytometry experiments demonstrated that following 48 h treatment, DEX also increases surface PD-1expression significantly in both CD4+ and CD8+ T cells (Fig. 2d).

### Effect of DEX on PD-1 mRNA and protein level

To determine whether DEX affected PD-1 expression at the mRNA level, mouse T cells were stimulated for 2 days with or without DEX (10^−7^ M). As shown in Fig. 2e, quantitative RT-PCR determined that PD-1 mRNA was increased significantly by DEX. The mean augmentation of PD-1 mRNA by DEX-treated group was determined to be more than fourfold over that of DEX-untreated group.

Western blot analysis showed that addition of DEX to activated T cells resulted in a significant increase of PD-1 protein (Fig. 2f). These data showed that DEX-induced augmentation of PD-1 expression by flow cytometry analysis correlates with an augmentation of both PD-1 mRNA and PD-1 protein.

### RU486 overcomes DEX-induced upregulation of PD-1 expression

GCs mediate their well-defined transcriptional effects via a cytoplasmic glucocorticoid receptor (GR) which belongs to the nuclear receptor superfamily. In the cytoplasm, GCs interact with GR, leading to nuclear translocation of the activated receptor and subsequent binding to negative or positive GR responsive elements (GREs) in the promoter regions of target genes [19]. RU486 has been described as a GR antagonist and was used to block activities of GCs mediated through GR.

**Fig. 3:**
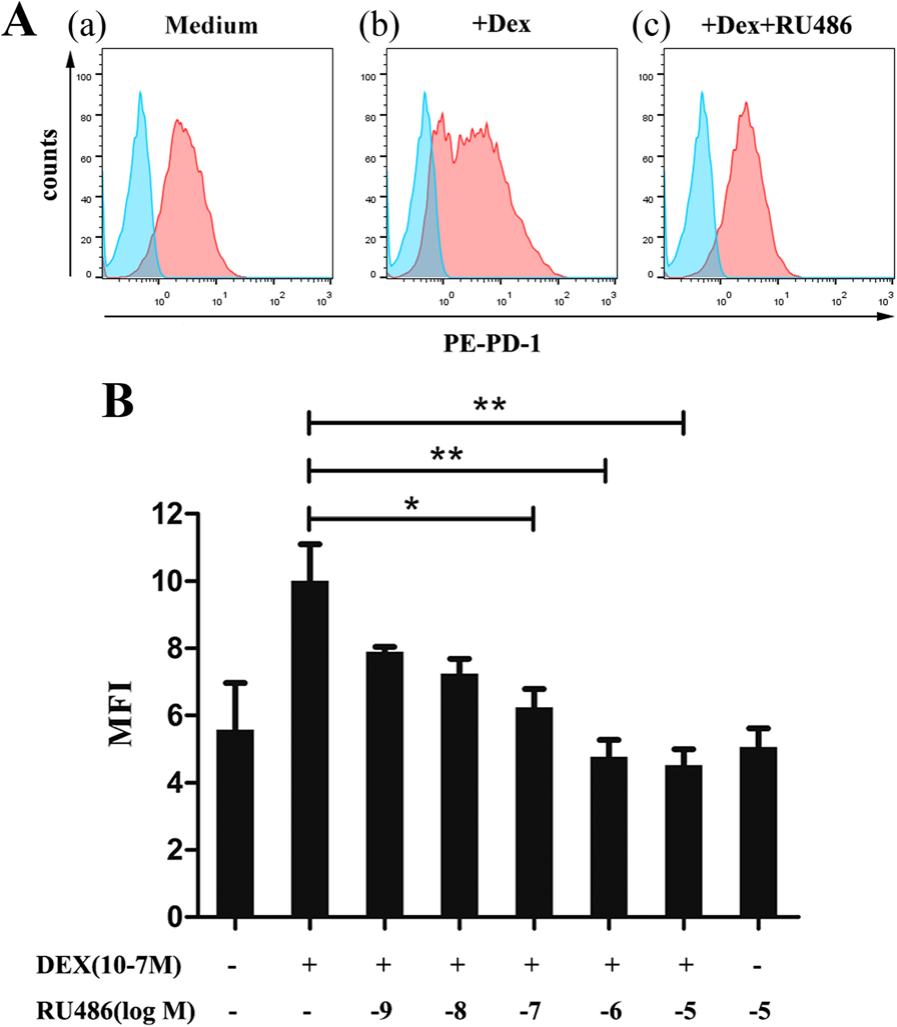
RU486 inhibits DEX-mediated increase of PD-1 expression. **a** Cells were cultured for 48 h in (a) medium (b) with 1 × 10^−7^ M DEX or (c) 1 × 10^−7^ M DEX plus 1 × 10^−5^ M RU486 for 48 h. Cells were stained with isotype control (cyan histograms) or PE-labeled anti-PD-1 antibody (red histograms). **b** Cells were cultured for 48 h with 1 × 10^−7^ M DEX or 1 × 10^−7^ M DEX plus different doses of RU486 or 1 × 10^−7^ M RU486 alone. Data is presented as the mean ± SEM of three experiments. *p < 0.05. **p < 0.01

In order to determine if the DEX-induced increase in PD-1 expression is due to specific glucocorticoid-GR interaction, the ability of RU486 to block the hormone effect was studied. Splenocytes were stimulated in the presence of DEX (1 × 10^−7^ M) alone or DEX plus various doses RU486 (1 × 10^−9^–10^−5^ M). The results showed that the augmentation of PD-1 expression by DEX was dose-dependently inhibited by RU486 (Fig. 3), indicating that the effect of DEX on PD-1 is mediated through the GR.

### Different T cell subsets show different sensitivity to DEX-induced upregulation of PD-1 expression

**Fig. 4:**
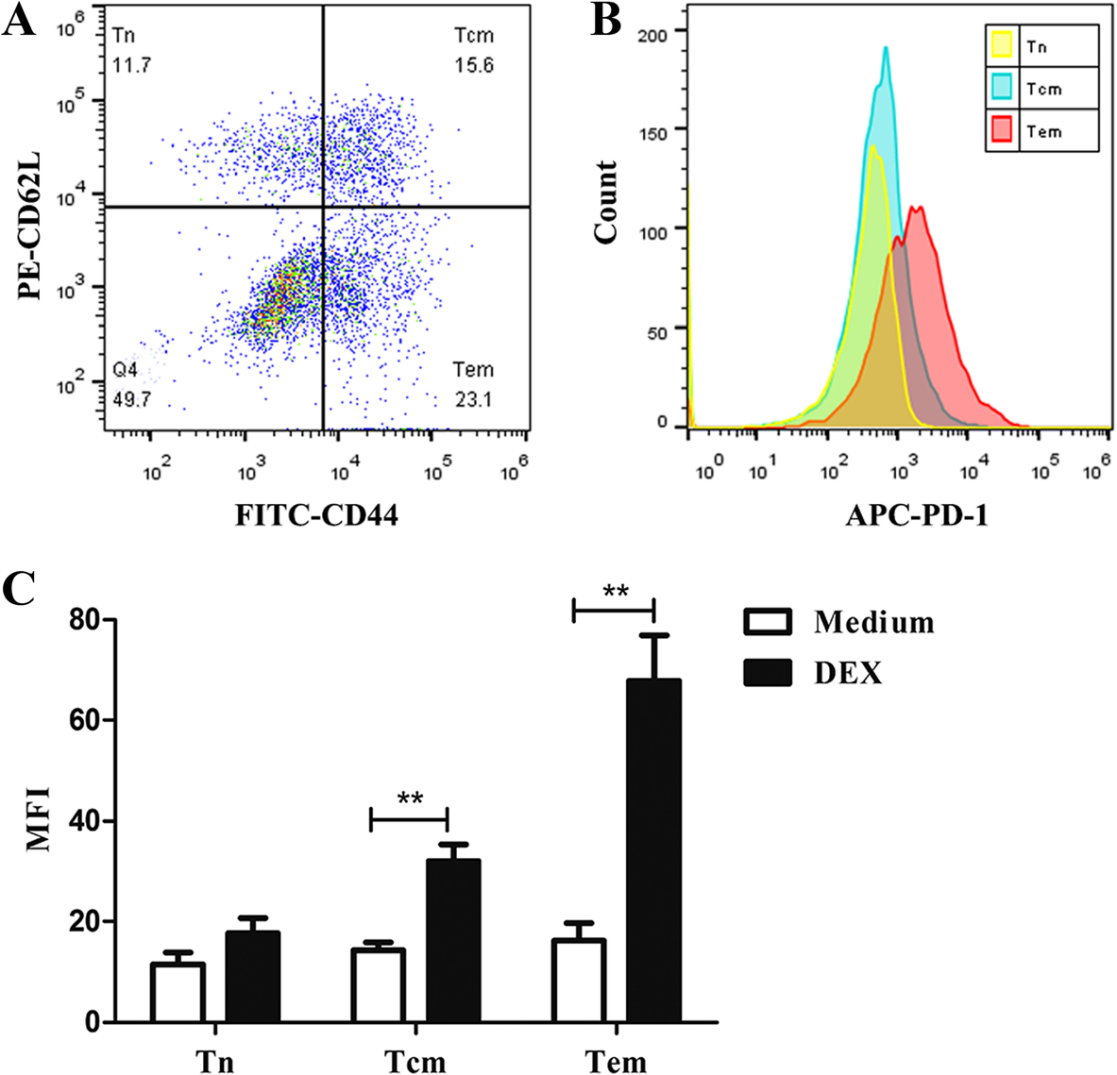
Different T cell subsets differ in their expression of PD-1 and sensitivity to DEX. Cells were incubated with anti-CD3/CD28 beads for 48 h in the absence or presence of 1 × 10^−7^ M DEX. **a** Based on the expression of CD44 and CD62L, four subsets were gated: CD44^−^CD62L^+^ (naïve T cells, Tn), CD44^+^CD62L^+^ (central memory T cells, Tcm), CD44^+^CD62L^−^ (effector memory T cells, Tem) and CD44^−^CD62L^−^ (double negative T cells). **b** Representative flow cytometry histograms show PD-1expression differs in Tn, Tcm and Tem, with Tem expressing the highest level and Tn the lowest. **c** Tcm, Tem are more susceptible to DEX-induced upregulation of PD-1. Data is presented as mean ± SEM of three experiments. **p < 0.01

T cells can be divided into several different subsets according to their functional and phenotypic features. According to the expression of the differentiation markers CD44 and CD62L, T cells can be divided into three subsets. A naïve phenotype (naïve T cells, Tn) is characterized by expression of low levels of CD44 and high levels of CD62L (CD44^lo^ CD62L^hi^). When naïve T cells react to antigen during the immune response, a small proportion of the responding cells survives to form antigen-specific memory T cells (Tm); these cells are typically CD44^hi^, with some of the cells being CD62L^hi^ (central memory T cells, Tcm) and others being CD62L^lo^ (effector memory T cells, Tem) [20]. Flow cytometry results showed that the expression of PD-1 had a significant difference between them, with Tem expressing the highest level and Tn the lowest (Fig. 4). Interestingly, these three subsets we gated are differentially modulated by DEX in terms of PD-1 expression. DEX induced a strong upregulation of PD-1 on Tcm and Tem, but a weaker upregulation on Tn (Fig. 4).

### Effect of hydrocortisone (HC) on PD-1 expression in mouse T cells

**Fig. 5:**
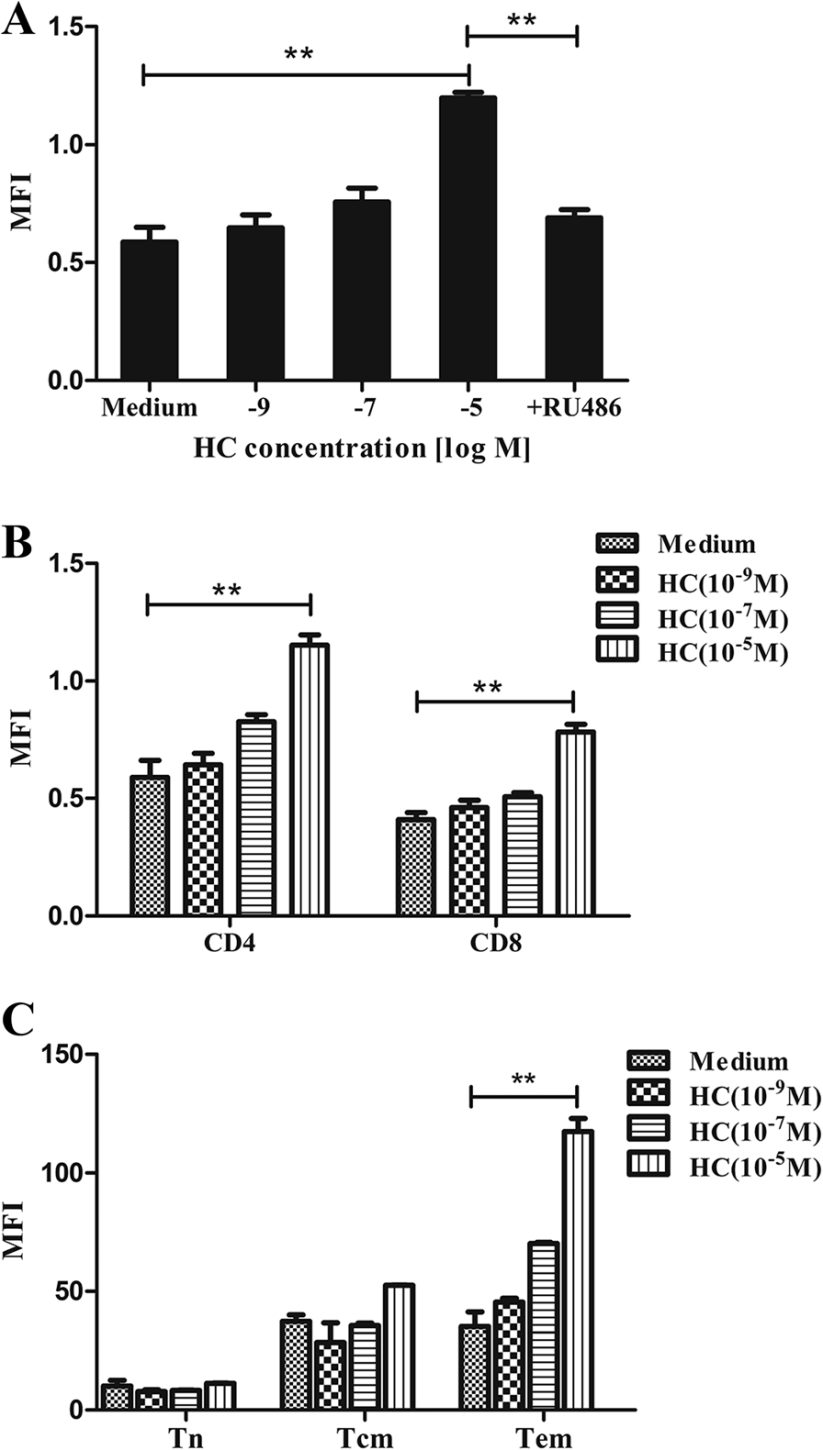
Effects of HC on PD-1 expression in mouse T cells. **a** Activated mouse T cells were cultured in medium, HC (10^−9^, 10^−7^, 10^−5^ M) or HC (10^−5^ M) plus RU486 (10^−5^ M) for 48 h. Surface PD-1 expression was increased in a dose-dependent manner and this effect was inhibited by RU486. **b** PD-1 expression was increased in a dose-dependent manner both in CD4+ and CD8+ T cells. **c** Tem are more susceptible to HC-induced upregulation of PD-1. Data is presented as mean ± SEM of three experiments. **p < 0.01

In order to assess whether less potent GCs have the same effect, we treated activated mouse T cells with hydrocortisone (HC) for 48 h. Similar to DEX, results showed that HC also increases surface PD-1 expression in a dose-dependent manner and this effect was inhibited by RU486 (Fig. 5a). Since HC is less potent than DEX, we noted that only at high dose with a concentration of 10^−5^ M can HC induce this effect significantly. Lower concentrations of HC caused weak effect of this kind. Thus we concluded that the effect of GCs on PD-1 expression depends on their potency and dose. At the same dose, more potent GCs such as prednisone and DEX could exert greater effect than less potent GCs like cortisol. Besides, we observed that the effect of HC on different subsets in terms of PD-1 were all in line with DEX (Fig. 5b, c).

### Effects of DEX on the function and apoptosis of activated T cells

GCs are potent anti-inflammatory and immunosuppressive agents. Their immunosuppressive effects have been ascribed to their modulation of gene transcription, whereby they inhibit the production of a number of cytokines (CKs), such as interleukin-2 (IL-2), interferon-γ (IFN-γ), and tumor necrosis factor-α (TNF-α) [21]. In vitro studies have also shown that the engagement of PD-1 by PD-L1 or PD-L2 inhibited TCR-mediated T cell proliferation and cytokine production including IL-2, IFN-γ and TNF-α [17, 22].

**Fig. 6:**
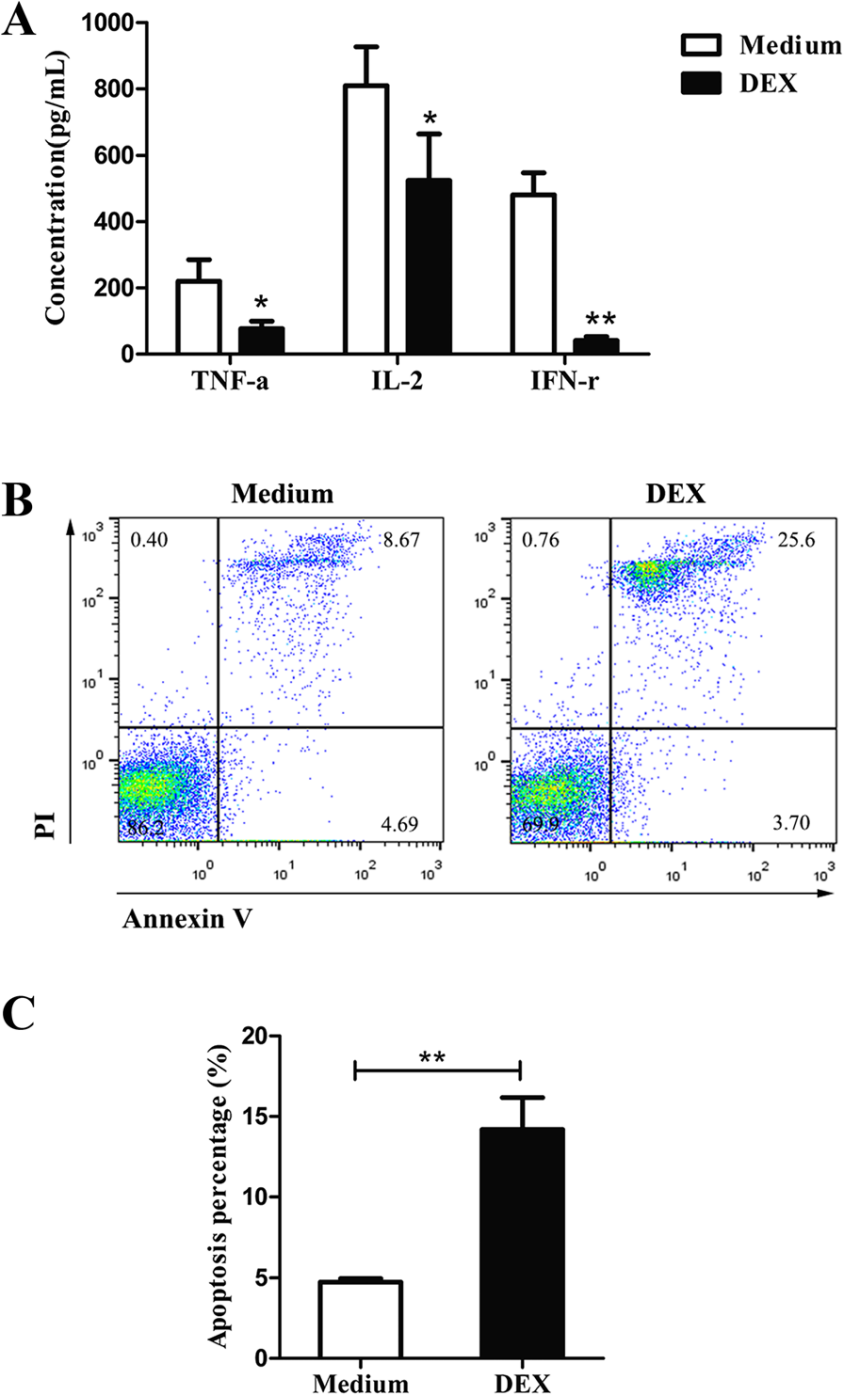
Effects of DEX on the function and apoptosis of activated T cells. Activated mouse T cells were cultured with DEX (10^−7^ M) or medium for 48 h. **a** Culture supernatants were collected and assayed for IL-2, TNF-α and IFR-γ. Data are shown as the mean values (±SEM) for triplicate cultures for each experiment. The results are from two independent experiments. Statistically significant differences between medium alone and DEX-treated groups was evaluated at *p < 0.05 or **p < 0.01. **b** Following 48 h incubation, cells were analyzed for apoptosis by FITC-Annexin V and PI staining by flow cytometric analysis. Annexin-V-, PI- cells were live cells, Annexin-V+, PI- cells were early apoptotic cells, Annexin-V+, PI+ cells were late apoptotic and dead cells were PI positive. **c** The percentages of apoptotic cells were labeled as Annexin V+ cells. Data is presented as mean ± SEM of three experiments. **p < 0.01

To validate the effect of DEX on the function of activated T cells, culture supernatants of mouse T cells activated for 48 h with DEX (10^−7^ M) or not were assayed for the production of cytokines IL-2, IFN-γ, TNF-α by ELISA. Results shown in Fig. 6a displayed significantly (p ≤ 0.01) decreased levels of IL-2, IFN-γ and TNF-α in DEX-treated group compared to DEX-untreated group, suggest that DEX suppressed the production of cytokines and inhibited the function of activated T cells.

Since DEX is known to be an apoptotic agent, we also evaluated the apoptosis at different times (6, 12, 24, 48 h) of treatment. Results showed significant higher levels of apoptosis in DEX-treated cells (Fig. 6b, c).

## Discussion

Activation of the immune system is recognized as an important treatment strategy against cancer. However, GCs suppress the immune system and many studies have shown that immunosuppression can exacerbate the metastatic process and accelerate tumor growth [23, 24]. The present study was designed to examine the effects of GCs on PD-1 expression during the course of T cell activation. To explore the possible effects, we first address the biological features of PD-1. Using flow cytometry analysis, we demonstrated that surface expression of PD-1 on activated T cells peaked on day 2 and returned to background levels after 6 days. This dynamic expression was in agreement with previous studies [15, 16].

Next we used DEX as a model glucocorticoid. Our data demonstrate for the first time that DEX could enhance the expression of PD-1 during activation of T cells. This finding is of great importance for the widespread use of DEX in anti-cancer therapy as well as the crucial role of PD-1 in tumor immunity. As an immune checkpoint, PD-1 serve to suppress the activity of T cells and dampen the immune response. Tumors can exploit this checkpoint and facilitate tumor progression. This significance sheds light on the development of monoclonal antibody targeting PD-1 against many cancers. Two antibodies that block PD-1, pembrolizumab (Keytruda) and nivolumab (OPDIVO), have been approved to treat advanced or inoperable melanoma and the clinical responses are encouraging [25, 26]. Therefore, the upregulation of PD-1 induced by DEX very likely protects cancer cells from being detected and eradicated by immune system, resulting in faster tumor growth and poor prognosis in anti-cancer therapy. We also validated that DEX could suppress T cell functions via inhibition of cytokines production such as IL-2, IFN-γ, TNF-α and induction of apoptosis of T cells. In this respect, administration of DEX to cancer patients may do harm to cancer patients.

Besides, we found that both DEX and HC enhances PD-1 expression in a dose-dependent manner, indicating that the dose of GCs administered to patients is of great importance. Widely used in high doses in the therapy of leukemia and lymphomas, GCs are highly suspicious to impair the effect of chemotherapy in the clinical setting of such cancers. For example, in the R-CHOP regimen for DLBCL, high dose of prednisone may suppress the activity of NK cells and T cells as well as the specific antitumor immune response triggered by rituximab, thus unfavorable to the protective immunity. In this way we suggest that alternative drugs should be applied instead of GCs or use low dose of GCs in anti-cancer therapy. We should minimize our application of glucocorticoids, especially high dose of glucocorticoids. Our data also showed the effects induced by DEX were completely inhibited by RU486, indicating that the effect of DEX on PD-1 is mediated through GR. A better understanding of the mechanism elucidating these effects is needed and will require further research into the downstream signals.

It should be noted that memory T cells were the subpopulation more susceptible to DEX-induced upregulation of PD-1. The higher sensitivity on memory T cells detected in this study may result in their faster apoptosis via PD-1, which helps explain why treatment with DEX could induce a significant decrease of memory T cells accompanied by a relative increase of naïve T cells (data not shown). In fact, memory T cells have more powerful potent to recognize tumor antigens and to exert immune response against cancers than naïve T cells. In this sense, the selective effect on memory T cells of DEX further validate its immunosuppressive role.

## Conclusion

Our findings provide evidence that dexamethasone could suppress immune response by enhancing PD-1 which may result in faster tumor growth and poor prognosis in the clinical setting of anti-cancer therapy. We strongly suggest that GCs have negative influence on anti-cancer therapy despite their benefits for patients. It is necessary to minimize our application of glucocorticoids, especially high dose of glucocorticoids in anti-cancer therapy. Further studies on the dual role of GCs are needed.

## Abbreviations

PD-1: Programmed cell death 1
CD: Cluster of differentiation
GCs: Glucocorticoids
DEX: Dexamethasone
HC: hydrocortisone
GR: Glucocorticoid receptor
RU486: Mifepristone
DLBCL: Diffuse large B-cell lymphoma
CTLA-4: Cytotoxic T lymphocyte associated antigen 4
PD-L1: Programmed cell death ligand 1
Percp-cy5.5: Peridinin chlorophylla protein cyanine 5.5 complexes
PE: Phycoerythrin
FITC: Fluorescein isothiocyanate
APC: Allophycocyanin
NKC: Natural killer cells
DC: Dendritic cells
MFI: Mean fluorescence intensity
GRE: glucocorticoid receptor responsive elements
Tn: Naïve T cells
Tm: Memory T cells
Tem: Effector memory T cells
Tcm: Central memory T cells
IL-2: Interleukin-2
IFN-γ: Interferon-γ
TNF-α: Tumor necrosis factor-α
